# Acute Mechanical Performance of Magmaris vs. DESolve Bioresorbable Scaffolds in a Real-World Scenario

**DOI:** 10.3389/fcvm.2021.696287

**Published:** 2021-06-14

**Authors:** Niklas F. Boeder, Oliver Dörr, Tim Koepp, Florian Blachutzik, Stephan Achenbach, Albrecht Elsässer, Christian W. Hamm, Holger M. Nef

**Affiliations:** ^1^Medical Clinic I, University Hospital of Giessen, Giessen, Germany; ^2^Medical Clinic 2, University Hospital of Erlangen, Erlangen, Germany; ^3^Department of Cardiology, University Hospital of Oldenburg, Oldeburg, Germany; ^4^Department of Cardiology, Kerckhoff Heart and Thorax Center, Bad Nauheim, Germany; ^5^German Centre for Cardiovascular Research, RheinMain Chapter, Frankfurt am Main, Germany

**Keywords:** bioresorbable scaffold, optical coherance tomography, Magmaris sirolimus-eluting bioresorbable scaffold, coronary heart disease, coronary imaging

## Abstract

**Background:** After the bioresorbable PLLA-based vascular scaffold (Absorb BVS) was taken from the market due to its high adverse event rates, a magnesium-based scaffold (Magmaris) was introduced.

**Objective:** To compare the acute performance of the sirolimus-eluting magnesium alloy Magmaris scaffold with that of the novolimus-eluting PLLA-based DESolve scaffold in terms of appropriate scaffold deployment using optical coherence tomography (OCT).

**Methods and Results:** Data from the final OCT pullback of 98 patients were included (19 Magmaris, 79 DESolve) and analyzed at 1-mm intervals. The following indices were calculated: mean and minimal area, residual area stenosis, incomplete strut apposition, tissue prolapse, eccentricity index, symmetry index, strut fracture, and edge dissection. OCT showed a minimum lumen area for Magmaris vs. DESolve of 6.6 ± 1.6 vs. 6.0 ± 1.9 (*p* = 0.06). Scaffolds with residual area stenosis >20% were predominantly seen in the DESolve group (15.8 vs. 46.8%; *p* = 0.01). The mean eccentricity index did differ significantly (0.74 ± 0.06 vs. 0.63 ± 0.09; *p* < 0.001). No fractures were observed for Magmaris scaffolds, but 15.2% were documented for DESolve BRS (*p* < 0.001). Incomplete scaffold apposition area was significantly higher in the DESolve group (0.01 ± 0.02 vs. 1.05 ± 2.32 mm^2^; *p* < 0.001).

**Conclusion:** This is the first study to compare the acute mechanical performance between Magmaris and DESolve in a real-world setting. The acute mechanical performance of Magmaris BRS seems to be superior to that of DESolve BRS, whereas OCT showed a good acute mechanical performance for both BRS in terms of generally accepted imaging criteria.

## Introduction

As the latest innovation in coronary stent therapy, the bioresorbable scaffold (BRS) was rapidly embraced by the interventional cardiologist community due to its potential for long-term benefits and for overcoming limitations inherent to existing drug eluting metallic stents (DES) (1). The absence of permanent metal in the treated vessel wall was seen as an advantage regarding some of the issues that are still unresolved despite improvements in existing DES platforms. BRS were conceived with the concept of offering transient vessel support in that they dissolve after several years. Hereby, they allow and support the restoration of vasomotor function and at the same time facilitate future surgical revascularization (1–3). However, current BRS have been shown to have important limitations including lower radial strength, lower expansion capabilities, and higher rates of scaffold thrombosis when compared with DES (4–6). Ultimately, the most widely investigated poly-L-lactic acid (PLLA) BRS, the Absorb BVS (Abbott Vascular, Santa Clara, CA, USA), was taken from the market. More recently, a magnesium-based scaffold was introduced, the Magmaris (Biotronik AG, Bülach, Switzerland). Mechanical properties of the alloy are closer to permanent metallic DES, and this may be reflected in its improved properties over the PLLA scaffolds. In fact, Waksman et al. (7) were able to demonstrate that the Magmaris is significantly less thrombogenic compared with the Absorb BVS in an *ex vivo* porcine arteriovenous shunt model, and available outcome data are thus far promising (8). Hence, we sought to investigate the acute mechanical performance of Magmaris in comparison with the PLLA-based DESolve BRS (Elixir Medical Corporation, Sunnyvale, CA, USA) in patients who were treated in a real-world scenario.

## Materials and Methods

### Scaffold Devices

The *Magmaris BRS* is a sirolimus-eluting magnesium alloy scaffold (9). The alloy degrades to magnesium hydroxide, magnesium phosphate, and amorphous calcium phosphate. Approximately 95% of the magnesium is resorbed at 12 months. Drug dose is 1.4 μm/mm^2^ and strut thickness is 150 μm. The maximum expandable diameter is 0.6 mm over the nominal diameter at 10 atm. There are two radiopaque tantalum markers at each end (10).

The *DESolve BRS* is a novolimus-eluting PLLA-based scaffold with a resorption time of 1–2 years. The design comprises sinusoidal hoops with nine peaks and valleys and three connecters per hoop. Strut thickness is 150 μm (11). It can be overexpanded up to 0.5 mm above the nominal diameter. Furthermore, the DESolve BRS is able to self-correct for minor malappositioning (12).

### Patient Cohort

Consecutive patients undergoing percutaneous coronary intervention (PCI) with either Magmaris or DESolve BRS irrespective of their clinical presentation were enrolled in this retrospective study. The index procedure was between January 2014 and July 2017 at the University of Gießen.

All patients gave written informed consent. The investigation conforms to the principles outlined in the declaration of Helsinki and was approved by the local ethics committee.

### Procedure and OCT Analysis

PCI was performed in accordance with standard clinical practice. The radial approach was favored, using a six French guiding catheter. If necessary, access was switched to femoral. Patients were administered unfractionated heparin at 70 U/kg body weight at the beginning of the procedure. Prior to lesion preparation patients were administered intracoronary nitroglycerine. Lesion preparation was initiated with pre-dilatation utilizing a non-compliant balloon that corresponded 1:1 to vessel size. The use of a debulking device was left to the operator's discretion. BRS was chosen to be sized 1:1 with respect to the vessel diameter. Its deployment was accomplished by slowly inflating the scaffold balloon (1 atm over 10 s, 2 atm over 10 s, and then 2 s per atm). The final pressure was maintained for 20 s. Post-dilatation was performed with a non-compliant balloon in accordance with the maximum expansion limits of the BRS.

Frequency domain optical coherence tomography (OCT) was performed using the Ilumen Optis system (St. Jude Medical, Inc., Minneapolis, MN, USA). Pullbacks were performed at 36 mm/s during contrast injection at a rate of 3–5 ml/s: after having placed the imaging catheter distally to the lesion, the pullback was recorded until either the guiding catheter was reached or the maximum pullback length was completed. A second sequential pullback was combined to image the whole lesion, if necessary. Data from the final pullback just before the end of the procedure were used for the offline analysis in this study.

**Figure 1:**
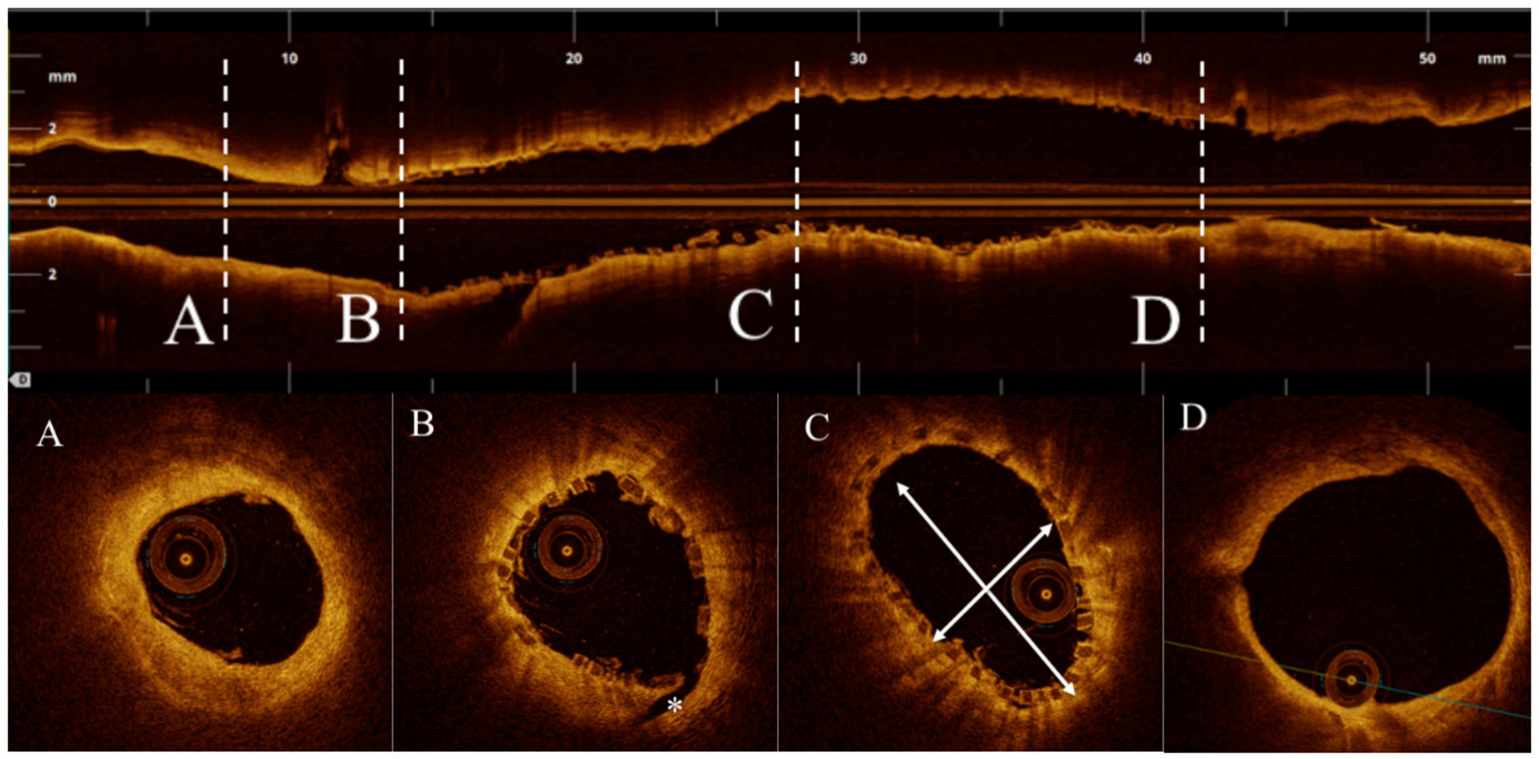
**(A)** Distal reference vessel area (DRVA) = 3.77 mm^2^. **(B)** Asterisk indicating a distal edge dissection. **(C)** Cross section with minimum eccentricity index (minimum/maximum diameter) = (2.29/3.96 mm) = 0.57. **(D)** Proximal reference vessel area (DRVA) = 11.59 mm^2^. Reference vessel area (RVA) = (PRVA + DRVA)/2 = (11.59 + 3.37 mm^2^)/2 =7.48 mm^2^.

**Figure 2:**
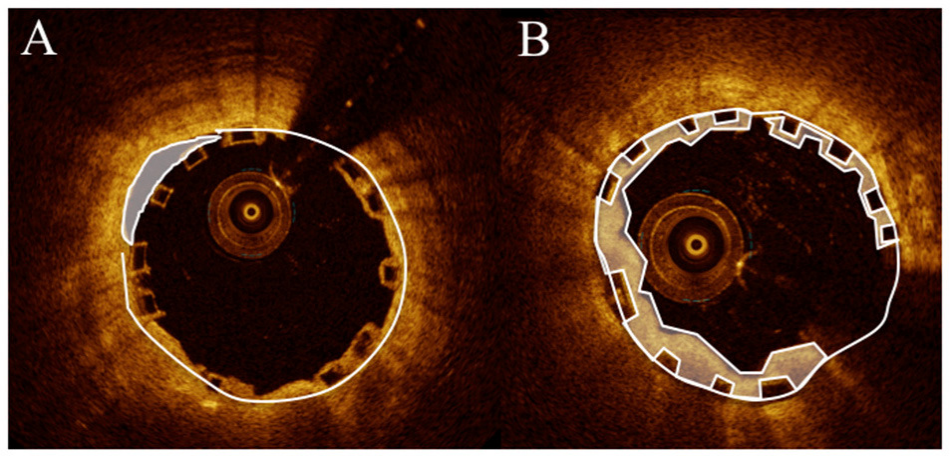
**(A)** Malapposition (lumen area – scaffold area) = (6.73 – 6.45 mm^2^) = 0.28 mm^2^. **(B)** Prolaps area (scaffold area – lumen area) (4.95 cm^2^ – 3.38 mm^2^) = l.57 mm^2^.

OCT measurements were performed offline using the LightLab Imaging workstation (St. Jude Medical, Inc.). The pullback was divided into cross-sections at 1-mm intervals within the stented lesion and 5 mm proximally and distally to the scaffold (Figures 1, 2). The following quantitative parameters were determined manually: the percentage of incomplete strut apposition (ISA) at 1-mm intervals, calculated as a percentage of the total number of malapposed struts divided by the total number of struts; the ISA area; the tissue prolapse area, defined as the projection of tissue into the lumen between struts; residual area stenosis (RAS) calculated as [1 – minimum lumen area (MLA)/reference vessel area (RVA)]; the eccentricity index, computed as the ratio between the minimum and maximum diameters; the symmetry index, defined as the difference between maximum scaffold diameter and minimum scaffold diameter divided by the maximum scaffold diameter. If no meaningful value for proximal or distal RVA was obtained, the largest luminal cross-sectional area at the distal or proximal end of the scaffold was used. An edge dissection was defined as any disruption of the vessel luminal surface at the edges of the scaffold with a visible flap (>300 μm). A scaffold fracture was assumed if isolated struts were observed to be unopposed within the scaffold lumen or if struts were stacked and discontinuities were present.

Quantitative coronary angiography (QCA) analysis was carried out with the help of offline QCA software (Medis Suite 3.2.36.2, Medis Medical Imaging, Leiden, The Netherlands). The following parameters were assessed during *post-hoc* analysis: reference vessel diameter (RVD) through automatic interpolation, minimum lumen diameter (MLD), percentage area stenosis (AS), and lesion length.

### Statistics

Statistical analysis was performed using IBM SPSS Statistics (SPSS Statistics 23, IBM Deutschland GmbH, Ehningen, Germany). Continuous variables with normal distribution are expressed as mean and standard deviation; categorical variables are given as number and percent. Chi-square and Fisher's exact test were used for comparison of categorical variables, and Student's *t*-test or the Wilcoxon rank-sum test was applied for continuous variables. *p* < 0.05 were considered statistically significant.

## Results

A total of 98 patients were enrolled in this study. Nineteen patients were implanted with a Magmaris BRS and 79 patients were treated with a DESolve BRS. More female patients were enrolled in the Magmaris BRS group (68.4 vs. 36.7%; *p* = 0.01). Patients in the DESolve BRS group had less frequently been treated by percutaneous intervention (57.9 vs. 26.6%; *p* = 0.002). Otherwise, patients did not differ significantly with respect to further baseline characteristics, including age (62.0 ± 8.1 vs. 61.7 ± 8.9; *p* = 0.81) and clinical presentation (Table 1).

**Table 1:** Baseline characteristics.

	**Magmaris BRS (*n* = 19)**	**DESolve BRS (*n* = 79)**	***p***
Age (years)	62.0 ± 8.1	61.7 ± 8.9	0.81
Female sex (%)	68.4	36.7	0.01^*^
Hypertension (%)	78.9	91.1	0.32
Hyperlipoproteinemia (%)	63.2	65.8	0.95
Diabetes mellitus (%)	10.5	19.0	0.42
Current Smoker (%)	42.1	63.4	0.21
Family history (%)	36.8	27.8	0.57
Prior PCI (%)	57.9	26.6	0.002^*^
Prior MI (%)	36.8	27.8	0.28
Left ventricular ejection fraction	55.7 ± 8.7	55.1 ± 9.9	0.99
**Clinical indication**			0.36
Stable angina (%)	66.7	57.0	
STEMI (%)	0	15.2	
NSTEMI (%)	11.0	7.6	
Unstable Angina (%)	22.2	20.3	
**Number of vessels diseased**			0.89
1 (%)	16.7	21.5	
2 (%)	27.8	26.6	
3 (%)	55.6	51.9	

*PCI, percutaneous coronary intervention; MI, myocardial infarction; STEMI, ST-elevation myocardial infarction; NSTEMI, non-ST-elevation myocardial infarction; BRS, bioresorbable scaffold*;

* *indicating clinical significance*.

Lesions were located in the right coronary artery for most of the patients. Overall, there was no difference with regard to the vessel that was treated between both groups (Table 2). Most of the lesions were of low complexity according to AHA/ACC lesion classification. QCA analysis showed no difference between the two groups with respect to reference vessel diameter (2.4 ± 0.7 vs. 2.5 ± 0.6 mm; *p* = 0.57), area stenosis (68 vs. 74.6%; *p* = 0.86), and lesion length (12.2 ± 4.5 vs. 10.4 ± 4.8 mm; *p* = 0.10).

**Table 2:** Angiographic and QCA lesions characteristics.

	**Magmaris BRS (*n* = 19)**	**DESolve BRS (*n* = 79)**	***p***
**Target vessel**			0.78
LAD (%)	31.6	35.4	
LCX (%)	15.8	22.8	
RCA (%)	47.4	41.8	
**AHA/ACC lesion classification**			0.74
Type A (%)	36.8	28.6	
Type B1 (%)	26.3	39.0	
Type B2 (%)	21.1	20.8	
Type C (%)	15.8	11.7	
**QCA analysis**
RVD (mm)	2.4 ± 0.4	2.5 ± 0.6	0.57
MLD (mm)	1.1 ± 0.24	1.2 ± 0.45	0.86
AS (%)	68.0	74.6	0.08
Lesion length (mm)	12.2 ± 4.5	10.4 ± 4.8	0.10

*LAD, left anterior descending artery; LCX, left circumflex artery; RCA, right coronary artery; AHA/ACC, American Heart Association/American College of Cardiology; QCA, Quantitative coronary angiography; RVD, reference vessel diameter; MLD, mean lumen diameter; AS, area stenosis; BRS, bioresorbable scaffold*.

Lesion preparation started with a thorough pre-dilatation in all cases that were treated with Magmaris, and in the majority of DESolve BRS cases (100 vs. 94.9%; *p* = 0.33). There was no difference in maximum length and diameter of the balloon used for pre- and post-dilatation (Table 3) or its maximum inflation pressure. The implanted scaffolds were 3.1 ± 0.4 vs. 3.1 ± 0.4 mm (*p* = 0.19) in mean diameter and 18.9 ± 4.0 vs. 19.7 ± 5.7 mm (*p* = 0.68) in mean length. The deployment pressure applied did not deviate between patients that were treated with Magmaris or DESolve BRS (15.1 ± 1.9 vs. 13.9 ± 2.6 atm; *p* = 0.07). Post-dilatation was performed with a non-compliant balloon in all cases.

**Table 3:** Procedural characteristics.

	**Magmaris BRS (*n* = 19)**	**DESolve BRS (*n* = 79)**	***p***
Pre-dilatation (%)	100.0	94.9	0.33
Maximum diameter balloon pre-dilatation (mm)	3.2 ± 0.3	3.0 ± 0.4	0.06
Maximum pre-dilatation balloon length (mm)	16.6 ± 2.3	15.1 ± 3.7	0.33
Maximum pre-dilatation balloon inflation (atm)	16.5 ± 2.3	13.7 ± 3.1	0.09
Scaffold diameter (mm)	3.1 ± 0.2	3.1 ± 0.4	0.91
Scaffold length (mm)	18.9 ± 4.0	19.7 ± 5.7	0.68
Scaffold deployment pressure (atm)	15.1 ± 1.9	13.9 ± 2.6	0.07
Post-dilatation (%)	100.0	83.5	0.06
Maximum post-dilatation balloon diameter (mm)	3.5 ± 0.3	3.6 ± 0.6	0.41
Maximum post-dilatation balloon length (mm)	14.4 ± 2.7	15.2 ± 3.8	0.46
Maximum post-dilatation balloon inflation (atm)	15.4 ± 3.9	16.6 ± 3.5	0.29
Post-dilatation with NC	100.0	100.0	

*NC, non-compliant; BRS, bioresorbable scaffold*.

Results of the analysis of the final OCT pullbacks are summarized in Table 4. A total of 1,948 cross-sections were analyzed. Final mean and maximum scaffold diameters were similar between the two groups (mean: 3.1 ± 0.3 vs. 3.1 ± 0.4 mm; *p* = 0.30; maximum: 3.4 ± 0.3 vs. 3.4 ± 0.5 mm; *p* = 0.61). Likewise, the mean scaffold area and mean lumen area did not differ significantly between the two groups (Table 4). There was a significant difference in minimum scaffold diameter (2.9 ± 0.2 vs. 2.7 ± 0.4 mm; *p* = 0.01). The RAS was determined to be 13.5% in patients treated with Magmaris and 16.6% after DESolve implantation (*p* = 0.23). Overall, there were more DESolve scaffolds that showed a RAS >20% than Magmaris scaffolds (15.8 vs. 46.8%, *p* = 0.01). Furthermore, there was greater eccentricity as revealed by the mean eccentricity index (0.89 ± 0.2 vs. 0.77 ± 0.1; *p* < 0.001) and less symmetry according to the symmetry index (0.31 ± 0.08 vs. 0.42 ± 0.09; *p* < 0.001) in patients treated with a DESolve BRS. Malapposition was less frequently observed in the Magmaris group than in the DESolve group (0.03 vs. 2.3%; *p* < 0.001). OCT showed dissections more frequently in the DESolve group, and strut fractures were observed only in this group (Table 4).

**Table 4:** Optical coherence tomography findings.

	**Magmaris BRS (*n* = 19)**	**DESolve BRS (*n* = 79)**	***p***
Mean scaffold area (mm^2^)	7.9 ± 1.5	7.7 ± 2.1	0.54
Mean scaffold diameter (mm)	3.1 ± 0.3	3.1 ± 0.4	0.30
Minimum scaffold diameter (mm)	2.9 ± 0.2	2.7 ± 0.4	0.01^*^
Maximum scaffold diameter (mm)	3.4 ± 0.3	3.4 ± 0.5	0.61
Mean lumen area (mm^2^)	7.8 ± 1.9	7.5 ± 2.1	0.30
Minimum lumen area (mm^2^)	6.6 ± 1.6	6.0 ± 1.9	0.06
Percentage RAS (%)	13.5	16.6	0.23
Scaffold with RAS > 20% (%)	15.8	46.8	0.01^*^
Mean eccentricity index	0.89 ± 0.20	0.77 ± 0.10	<0.001^*^
Minimum eccentricity index	0.74 ± 0.06	0.63 ± 0.09	<0.001^*^
Symmetry index	0.31 ± 0.08	0.42 ± 0.09	<0.001^*^
**ISA**		
ISA area (mm^2^)	0.01 ± 0.1	1.05 ± 2.32	<0.001^*^
Percentage of malapposed struts (%)	0.03	2.3	<0.001^*^
Prolapse area (mm^2^)	0.0	4.7	<0.001^*^
Strut fracture (%)	0	15.2	0.07
Edge dissection			0.75
Proximal edge (%)	5.3	3.8	
Distal edge (%)	0	2.5	

*RAS, residual area stenosis; ISA, incomplete apposition area; BRS, bioresorbable scaffold*;

* *indication statistical significance*.

No adverse event occurred within the ensuing post-procedural period. Of note, no scaffold thrombosis was documented.

## Discussion

This is the first study to investigate the acute mechanical performance between the PLLA-based DESolve BRS and the magnesium-based Magmaris BRS by means of OCT in a real-world scenario. The principal findings are that OCT imaging criteria revealed a good acute mechanical performance for both BRS, but acute mechanical performance of the Magmaris BRS appeared to be superior to that of the DESolve BRS.

The patients enrolled in this study fulfilled the typical criteria for the implantation of BRS. They were relatively young and—especially in the DESolve BRS group—had a short history of coronary disease. Furthermore, the lesion complexity was predominantly simple and none of the cases involved a bifurcation or ostial lesion.

Imaging studies, which have predominantly used intravascular ultrasound (IVUS) to investigate and evaluate the acute and long-term clinical outcome in patients treated with either bare metal stents or first-generation DES, have found that a cross-sectional MLA <5.5 mm^2^ and/or an in-scaffold RAS >20% increases the risk of stent thrombosis. Patients treated in our cohort had a mean RAS of 13.5% in the Magmaris group and 16.5% in the DESolve group (*p* = 0.23). However, patients treated with the latter BRS more frequently had scaffolds with an RAS > 20% (15.8 vs. 46.8% of the respective group; *p* = 0.01). It should be noted that the periprocedural characteristics, particularly pre- and post-dilatation, did not significantly differ between groups. Moreover, there was only a tendency for the DESolve group to have a smaller MLA (6.6 ± 1.6 vs. 6.0 ± 1.9 mm^2^; *p* = 0.06), but this was not significant. As OCT tends to measure lower absolute areas than IVUS (13), the published reference values may not be entirely applicable for the assessment of adequate scaffold deployment; however, both groups showed values above the cut-offs.

Suwannasom et al. (14) found that BRS in general are more frequently associated with asymmetric and eccentric morphology compared with DES. Though, analysis of the final pullbacks shows a significant difference in post-procedural geometry between the two studied device types. In our study of the two BRS devices, the mean eccentricity index after implantation of Magmaris was significantly higher than that of DESolve (0.89 ± 0.2 vs. 0.77 ± 0.10; *p* <0.001). This finding is supported by the data of Abellás-Sequeiros et al. (15) and is of clinical relevance, as Suwannasom et al. were able to demonstrate that post-procedural asymmetry was independently associated with device-oriented composite endpoints. Therefore, our data suggest a more favorable acute result for the Magmaris group, although both groups met the imaging criteria that were evaluated in the MUSIC trial (16). Here, first-generation DES with an eccentricity index of 0.7 were associated with favorable angiographic results at the 6-month follow-up. The asymmetry can also be assessed by the symmetry index, with a value near zero indicating a symmetric structure throughout the entire length (17). Thus, the acute results after Magmaris implantation also appear to be superior regarding symmetry, showing less bending stiffness and greater flexibility.

Recently, malapposition was identified as a predictor of late and very late scaffold thrombosis in patients treated with BRS (18). One explanation is that malapposed struts may disrupt the laminar flow and activate platelets due to high shear stress (19), thereby contributing to the multifactorial etiology of scaffold thrombosis. Accordingly, event rates were reduced if the operator made use of intravascular imaging modalities to guide the implantation process of DES (20, 21). The prospective, multi-center PRAGUE 19 BRS study revealed 1.1% malapposed struts, much less than the 3.5% observed in the Absorb Cohort B study (22, 23). The rate of incomplete apposition in our cohort was negligible in the Magmaris group and 2.3% in the DESolve group (*p* < 0.001). It has previously been shown that post-dilatation to reduce malapposition can be done safely in patients undergoing BRS implantation (24); however, incomplete apposition of struts remained a problem in our DESolve group even after post-dilatation. Although the implantation and post-dilatation pressures did not differ between the groups, only patients treated with DESolve BRS showed fractures (0 vs. 15.2%; *p* = 0.07). This difference further underlines our impression that the PLLA-based platform is more prone to mechanical problems than the magnesium scaffold and suggests that the Magmaris has a greater expansion capacity and radial force. One can also speculate that underlying coronary plaque composition, morphology, and burden may have influenced this result. However, the dependency of expansion and eccentricity seems to be primarily a problem associated with the Absorb BVS (25) rather than the DESolve BRS (26). Barkholt et al. (27) performed a bench evaluation of the mechanical properties of the Magmaris BRS and compared them with those of DES and PLLA-based BRS. They demonstrated that the Magmaris was more resistant to strut fracture than the Absorb BVS. It had a larger crossing profile and similar radial and longitudinal strengths. While recoil after deployment was greater with Magmaris, all devices had similar diameters 120 min after 3.5-mm post-dilatation. Therefore, further randomized clinical trials are required to evaluate and identify the scaffolds that ultimately achieve the goal of matching the performance of the DES and overcoming their long-term limitations.

### Limitations

There are several limitations associated with this study. Its registry nature, with retrospective collection of patient data, has inherent limitations and the evidence provided should be seen as hypothesis generating. Furthermore, although the protocols used for lesion preparation, scaffold deployment, and post-dilatation were the same for all operators contributing to this study, their variable application due to the operator's decision may have affected the final acute mechanical result and cannot be excluded. Characteristic of a scaffold may not only be defined by its intrinsic properties but also lesion preparation and underlying morphological aspects. Therefore, it has to be taken into consideration that lesion preparation and post-dilatation numerically that differed between the two groups while not reaching statistical significance may have had an impact on acute mechanical result that cannot be excluded. Furthermore, the sample size of the study was small with few diabetic patients. Treated lesions affected the RCA in most cases while overall, there was no difference between the two groups regarding the distribution of the target vessel. Furthermore, the follow-up was limited to the post-procedural period, and it must be evaluated whether the acute findings translate into an improved long term clinical outcome.

## Conclusions

This is the first study that compares the acute mechanical performance of two different BRS that included Magmaris in a real-world setting. The Magmaris BRS seems to be a promising alternative to the DESolve BRS, especially after the bioresorbable PLLA-based Absorb BVS was taken from the market due to its high adverse event rates. The acute mechanical performance of the Magmaris BRS seems to be superior to the DESolve BRS, whereas OCT showed a good acute mechanical performance for both BRS in terms of generally accepted imaging criteria.

## Data Availability Statement

The datasets presented in this article are not readily available because of privacy reasons. Requests to access the datasets should be directed to the corresponding author.

## Ethics Statement

The studies involving human participants were reviewed and approved by University of Gießen. The patients/participants provided their written informed consent to participate in this study.

## Author Contributions

NB and HN designed the study and drafted the manuscript. NB, TK, FB, SA, AE, and CH contributed the data. All authors were involved in critically revising the manuscript.

## Conflict of Interest

The authors declare that the research was conducted in the absence of any commercial or financial relationships that could be construed as a potential conflict of interest.
